# Inflammation and depression: combined use of selective serotonin reuptake inhibitors and NSAIDs or paracetamol and psychiatric outcomes

**DOI:** 10.1002/brb3.338

**Published:** 2015-05-29

**Authors:** Ole Köhler, Liselotte Petersen, Ole Mors, Christiane Gasse

**Affiliations:** 1 Department P, Research Unit, Aarhus University Hospital RisskovRisskov, Denmark; 2 The Lundbeck Foundation Initiative for Integrative Psychiatric Research, iPSYCHAarhus, Denmark; 3 National Centre for Register-based Research, Aarhus UniversityAarhus, Denmark

**Keywords:** Antidepressants, depression, epidemiology, mood disorders, pharmacoepidemiology, pharmacotherapy

## Abstract

**Background:**

Nonsteroidal anti-inflammatory drugs (NSAIDs) and paracetamol have been shown to yield the potential of adjunctive antidepressant treatment effects to selective serotonin reuptake inhibitors (SSRIs); however, when investigating treatment effects of concomitant use, simultaneous evaluation of potential adverse events is important. The objective was thus to investigate treatment effectiveness and safety aspects of concomitant SSRI use with NSAIDs or paracetamol.

**Methods:**

Within a 25% random sample of the Danish population, we identified all incident SSRI users between 1997 and 2006 (*N* = 123,351). Effectiveness and safety measures were compared between periods of SSRI use only and periods of combined SSRI and NSAID or paracetamol use by applying Cox regression.

**Results:**

Among 123,351 SSRI users (follow-up: 53,697.8 person-years), 21,666 (17.5%) used NSAIDs and 10,232 (8.3%) paracetamol concomitantly. Concomitant NSAID use increased the risk of any psychiatric contact [Hazard rate ratio (95%-confidence interval): 1.22 (1.07; 1.38)] and with depression [1.31 (1.11; 1.55)]. Low-dose acetylsalicylic acid reduced the risk of psychiatric contact in general [0.74 (0.56; 0.98)] and with depression [0.71 (0.50; 1.01)]. Ibuprofen reduced the risk of psychiatric contacts [0.76 (0.60; 0.98)]. Concerning safety, paracetamol was associated with increased mortality [3.18 (2.83; 3.58)], especially cardiovascular [2.51 (1.93; 3.28)]. Diclofenac [1.77 (1.22; 2.55)] and the selective COX-2 inhibitors [1.75 (1.21; 2.53)] increased mortality risks.

**Conclusions:**

Concomitant use of SSRIs and NSAIDs occurred frequently, and effectiveness and safety outcomes varied across individual NSAIDs. Especially low-dose acetylsalicylic acid may represent an adjunctive antidepressant treatment option. The increased mortality risk of concomitant use of paracetamol needs further investigation.

## Introduction

An inflammatory state is associated with the etiology in subgroups of depressed individuals (Benros et al. 2013) and an increased risk of depression (Wium-Andersen et al. 2013). Thus, nonsteroidal anti-inflammatory drugs (NSAIDs) may be used as an adjunctive treatment option against depression in combination with antidepressants (Muller et al. 2006; Kohler et al. 2014) because of common anti-inflammatory mechanisms (Knights et al. 2010; Chavda et al. 2011), probably due to Cyclooxygenase-2 (COX-2) inhibition (Taler et al. 2007; Knights et al. 2010). Besides COX-2 inhibition, other potential mechanisms of NSAIDs concerning antidepressant properties include reduction in oxidative and nitrosative stress (Anderson et al. 2013), prevention of increase of proinflammatory cytokines (Casolini et al. 2002) and increment of central serotonin levels (Sandrini et al. 2002). Also paracetamol may act via COX-2 inhibition (Hinz and Brune 2012), but only one study has investigated the possible adjunctive effects of paracetamol (Warner-Schmidt et al. 2011).

Findings from animal and human studies are partly contradictory. Some studies found adjunctive antidepressant effects for acetylsalicylic acid (ASA)(Brunello et al. 2006; Mendlewicz et al. 2006) and the selective COX-2 inhibitor celecoxib (Muller et al. 2006; Akhondzadeh et al. 2009; Abbasi et al. 2012; Na et al. 2013), mostly in combination with Selective Serotonin Reuptake Inhibitors (SSRIs). Conversely, NSAIDs and paracetamol have been observed to inhibit antidepressant efficacy of the SSRI citalopram in both animal studies and a post hoc analysis of the Sequenced Treatment Alternatives to Relieve Depression trial (STAR*D)(Warner-Schmidt et al. 2011). A subsequent clinical study found no association between NSAID use and modified antidepressant treatment outcome (Uher et al. 2012), whereas a re-analysis of the STAR*D data found an association of decreased antidepressant treatment effect with NSAIDs in general, but not with ASA or COX-2 inhibitors (Gallagher et al. 2012). These discrepancies may be a result of differential effects of NSAIDs, COX-2 inhibitors and salicylates on antidepressant effects or confounding as pointed out by Almeida et al. (Almeida et al. 2010). Furthermore, potential adjunctive effects of NSAID add-on treatment may be counteracted by increased risks of gastrointestinal (GI) bleeding episodes (de Abajo and Garcia-Rodriguez 2008) and cardiovascular disease (CVD)(Schjerning Olsen et al. 2011), which were not considered in previous observational studies (Sandrini et al. 2002; Gallagher et al. 2012). Since antidepressants, NSAIDs and paracetamol are frequently used (Fosbol et al. 2008; Trifirò et al. 2013), it is important to further investigate whether adjunctive or adverse effects are most prominent at the population level, and beyond this to investigate the effect across the range of NSAIDs.

Therefore, we performed a longitudinal population-based cohort study of incident SSRI users with adjustment for important confounders, such as clinical and socio-demographic factors. We investigated whether or not treatment effects of SSRIs differed depending on concomitant use of different NSAIDs or paracetamol. Additionally, if concomitant NSAID or paracetamol use changed the rates of somatic adverse events and mortality.

## Materials and Methods

### Study population

The study population was identified from a 25% representative random sample of the Danish population (present population approximately 5.5 million inhabitants) using the Danish Civil Registration System (Pedersen 2011). Due to data access regulations, it was not possible to access the entire population. Prescription drug use was assessed by linkage to the Danish National Prescription Registry (Kildemoes et al. 2011), which contains detailed information on each drug dispensed, but not on the indication of treatment or dosage information. We identified incident users of SSRIs (Anatomical Therapeutic Chemical (ATC) code: N06AB) between January 1, 1997, and December 31, 2006 without antidepressant use in the year preceding the date of SSRI treatment initiation (index date). Each individual was only included once.

### Assessment of NSAID use

Prescriptions for NSAIDs (ATC-codes M01A and N02BA) were identified for each individual after the index date and during the preceding year. Low-dose ASA (B01AC06) was included because it has shown additional antidepressant effects (Mendlewicz et al. 2006). NSAIDs were classified according to their selectivity with regard to COX-1/COX-2 inhibition (Knights et al. 2010): salicylates (i.e., acetylsalicylic acid, diflunisal, salicylamid, low-dose ASA) due to their irreversible acetylation of COX-1 and -2 enzymes; nonselective NSAIDs (NS-NSAIDs, i.e., ibuprofen, naproxen, ketoprofen, dexibuprofen, tolfenamic acid, piroxicam) because of their nonselective inhibition of either COX-1 or -2; nonselective COX-inhibitors (NS-COX, i.e., diclofenac, etodolac, meloxicam, nabumetone) with a more pronounced COX-2 inhibition as compared to NS-NSAIDs; and selective COX-2 inhibitors (i.e., celecoxib, rofecoxib, valdecoxib, parecoxib, etoricoxib). Paracetamol (N02BE01) was assessed separately, as it may attenuate antidepressant treatment (Warner-Schmidt et al. 2011) and inhibit COX enzymes (Hinz and Brune 2012).

### Dose and duration of treatment

Prescription durations were calculated based on the prescribed number and strength of pills and the Daily Defined Dose (DDD)(World Health Organization 2008) for every drug because direct prescribed dosage information was not available. To account for compliance issues and differences in drug dosages across indications, the calculated treatment length was extended. For SSRIs, treatment duration of every prescription was extended by 7 days, and by 15 days for prescriptions on NSAIDs and paracetamol.

### Time at risk and follow-up

Follow-up started on the index date. Censoring occurred due to switching to another antidepressant drug, discontinuation of the first SSRI treatment episode, emigration, switching to another NSAID of a different class, after 3 years of follow-up or end of the study period, whatever came first. NSAID and paracetamol use was assessed time-varying during SSRI treatment: all individuals were for the entire follow-up period covered with SSRIs and could concomitantly be treated with NSAIDs or paracetamol.

### Outcome definition

Different measures were applied to evaluate effectiveness and safety of concomitant treatment with SSRIs and NSAIDs or paracetamol compared to SSRI monotherapy:

#### Effectiveness

1. Any hospital contact, including outpatient visits, due to any psychiatric disorder in the secondary healthcare system. Contacts were identified by linkage to the Danish Psychiatric Central Research Register (Mors et al. 2011). Diagnoses according to the International Classification of Mental and Behavioural Disorders, diagnostic criteria for research (ICD-10-DCR)(World Health Organization 1993).
2. A hospital contact, including outpatient visits, due to depression only in the secondary healthcare system (Mors et al. 2011) (ICD-10-DCR codes: F32 and F33).
3. Completed suicide and suicide attempts as two independent outcomes. Based on ICD-10 codes, completed suicide was identified from the Cause of Death Register (Helweg-Larsen 2011), attempted suicide from the Danish National Hospital Register (Lynge et al. 2011) or the Danish Psychiatric Central Research Register (Mors et al. 2011).

#### Safety

1. All-cause mortality (Pedersen 2011) and CVD and GI-mortality (Helweg-Larsen 2011).
2. Cause and frequency of any hospital contact, including outpatient visits, due to CVD and GI disorders (Lynge et al. 2011).

### Assessment of covariates: comorbidity and socio-demographic factors

Psychiatric disorders (Mors et al. 2011) were divided into: Disorders due to use of alcohol, disorders due to use of substance use, schizophrenia spectrum, bipolar disorder, depression, anxiety disorders, and all other remaining psychiatric disorders diagnoses (codes provided in Table S1). To account for somatic comorbidity (Lynge et al. 2011), we identified SSRI users suffering from a disorder of the musculoskeletal system or connective tissue due to the high comorbidity with pain-causing disorders (Manning and Jackson 2013). The eight revision of the ICD (ICD-8) was used until January 1, 1994, and from January 1994, ICD-10 has been used (Lynge et al. 2011; Mors et al. 2011). Furthermore, we applied the Charlson Comorbidity Index (Charlson et al. 1987), which includes 19 severe chronic somatic diseases such as heart disease, diabetes, and cancer. It has subsequently been validated and used in different diseases including nonmalignant (Almeida et al. 2010) and depression (Thygesen et al. 2011) and modified for use with the ICD-10 (Nuttall et al. 2006). The Charlson score was categorized into 0, 1, 2 and 3 or more depending on the number of comorbid somatic diseases.

Socio-demographic factors included gender, age, and educational level. Age-groups were divided into: 10–29, 30–49, 50–69, 70–89, and 90 years or older. Educational level covered: primary school, secondary school, vocational education, short, and medium higher education and a university degree (Jensen and Rasmussen 2011).

To adjust for other anti-inflammatory and analgesic medications, we identified use of corticosteroids (ATC-code H02A and H02B), anti-inflammatory/antirheumatic agents (M01B and M01C) and opioids (N02A) (23) within the preceding year. SSRIs and NSAIDs increase the risk of GI-bleeding (de Abajo and Garcia-Rodriguez 2008), why we identified prior use of H_2_-antagonists (A02BA), prostaglandins (A02BB), proton pump inhibitors (A02BC), helicobacter pylori eradication (A02BD), and drugs against gastro-esophageal reflux disease (A02BX).

### Statistical analyses

COX proportional hazard regression was performed to calculate hazard rate ratios (HRR) including 95-% confidence intervals (95-% CI). We used time since index date as underlying timescale with age as a continuous variable. Linear splines were applied and we included all potential covariates in the models: Gender; educational level; use of NSAIDs, paracetamol, other anti-inflammatory or GI-protective drugs within the year prior to index date; previous contacts with psychiatric and somatic disorders; Charlson Index Score; earlier suicide attempts; and start year with SSRI treatment.

In the primary analyses, we compared SSRI users to users of the combination therapy of SSRIs and NSAIDs or paracetamol. The secondary analyses comprised investigations of specific NSAID groups and single NSAIDs in combination with SSRIs as compared to SSRI monotherapy.

In addition, we performed several sensitivity analyses. The first consisted of repeating all analyses containing the calculated versus the extended prescription duration (7 days for SSRI and 15 days for NSAID and paracetamol users). In a second and third analysis, we performed age- and sex-specific subgroup analyses. As selective COX-2 inhibitors were available only during the period 1999–2006 and partially withdrawn in 2004, a fourth sensitivity analysis on these time-periods was performed (i.e., 1997–1998, 1999–2004, and 2005–2006). A fifth analysis consisted of all SSRI users suffering from a disease of the musculoskeletal system or connective tissue, as these patients are more inclined to take their prescribed NSAID medication. We further identified SSRI users with a diagnosis of rheumatoid arthritis (M00–M19) and or use of allopurinol and repeated all analyses. As prior psychiatric contacts could increase the risk for subsequent psychiatric contacts, we performed all analyses on SSRI users without any prior psychiatric contacts in a seventh analysis. In an eighth and final analysis, we performed a sensitivity analysis on incident NSAID and paracetamol users (i.e., without NSAID or paracetamol use in the preceding year).

To illustrate the cumulative hazard over time, we graphed cumulative incidences based on competing risk analyses, as death is a competing risk to all other outcomes, that is the risk for psychiatric contacts could be decreased because of an increased mortality risk.

## Results

We identified 123,351 individuals initiating SSRI treatment between 1997 and 2006 (total follow-up time: 53,697.8 person-years). Citalopram (58.2%) and sertraline (16.2%) were most frequently used. Of SSRI users, 21,666 (17.5%) redeemed prescriptions on NSAIDs (4.9% for low-dose ASA, 2.6% ASA, 5.1% ibuprofen, 2.4% diclofenac, 0.5% celecoxib, and 2% for others). 10,232 (8.3%) used paracetamol concomitantly. Table 2008 summarizes clinical and socio-demographic characteristics.

**Table 1:** Baseline characteristics among SSRI users and users of SSRIs in combination with NSAIDs or paracetamol

	Total *N* (%)	SSRI *N* (%)	SSRI& NSAID *N* (%)	SSRI & paracetamol *N* (%)
Total	123,351 (100)	91,456 (74.2)	21,666 (17.5)	10,232 (8.3)
Gender				
Female	75,616 (61.3)	55,790 (61.0)	13,024 (60.1)	6802 (66.5)
Male	47,735 (38.7)	35,666 (39.0)	8641 (39.9)	3428 (34.5)
Median age, IQR:	50.9 (35.4; 70.6)	46.5 (32.9; 64.2)	62.0 (45.3; 76.8)	76.0 (62.9; 83.8)
Age groups				
10–29	20,459 (16.5)	18,659 (20.4)	1700 (7.8)	109 (1.1)
30–49	39,494 (31.9)	33,386 (36.5)	5151 (23.8)	957 (9.4)
50–69	31,698 (25.7)	22,443 (24.5)	6658 (30.7)	2597 (25.4)
70–89	29,217 (23.9)	15,819 (17.3)	7545 (34.8)	5853 (57.2)
90+	2483 (2.0)	1158 (1.3)	611 (2.8)	714 (7.0)
Education				
Primary school	46,222 (37.5)	33,882 (37.0)	8398 (38.8)	3942 (38.5)
Secondary school	7136 (5.8)	6300 (6.9)	723 (3.3)	113 (1.1)
Vocational	32,637 (26.5)	25,037 (27.4)	5773 (26.6)	1827 (17.9)
Short higher	3460 (2.8)	2800 (3.1)	506 (2.3)	154 (1.5)
Medium higher	10,545 (8.6)	8585 (9.4)	1580 (7.3)	380 (3.7)
University	4523 (3.7)	3785 (4.1)	604 (2.8)	134 (1.3)
Previous disorders				
No contact	79,210 (64.2)	60,921 (66.6)	12,865 (59,4)	5424 (53.0)
Depression	3796 (3.1)	3003 (3.3)	561 (2.6)	232 (2.3)
Schizophrenia spectrum	2288 (1.9)	1810 (2.0)	282 (1.3)	196 (1.9)
Bipolar disorder	566 (0.5)	459 (0.5)	72 (0.3)	35 (0.3)
Anxiety	2744 (2.2)	2216 (2.4)	362 (1.7)	166 (1.6)
Alcohol	3620 (2.9)	2676 (2.9)	584 (2.7)	360 (3.5)
Drug	1759 (1.4)	1297 (1.4)	287 (1.3)	175 (1.7)
Other psychiatric^1^	12,868 (10.4)	9466 (10.4)	2152 (9.9)	1250 (12.2)
Musculoskeletal disease	29,537 (23.9)	19,259 (21.1)	6594 (30.4)	3684 (36.0)
Charlson score				
0	91,966 (74.6)	73,673 (80.1)	13,585 (62.7)	4708 (46.0)
1	13,341 (10.8)	7854 (8.6)	3492 (16.1)	1995 (19.5)
2	10,993 (8.9)	6394 (7.0)	2656 (21.3)	1943 (19.0)
3+	7051 (5.7)	3535 (3.9)	1932 (8.9)	1584 (15.5)
Prior NSAID use^2^				
No use	73,519 (59.6)	59,172 (64.2)	6403 (29.6)	1910 (18.7)
Any use	49,951 (40.5)	27,301 (35.8)	15,263 (70.4)	8322 (81.3)
Salicylates	17,806 (14.4)	5972 (10.7)	8247 (38.1)	3073 (30.0)
NS-NSAIDs	19,850 (16.1)	10,666 (13.4)	5780 (26.7)	2041 (19.9)
Non-selective COX	12,048 (9.8)	6013 (7.8)	3639 (16.8)	1451 (14.2)
Selective COX-2	3204 (2.6)	842 (1.5)	1165 (5.4)	714 (7.0)
Paracetamol	17,579 (14.3)	5231 (9.1)	4074 (18.8)	6720 (65.7)
Prior GI-drug use				
No prior use	104,699 (84.9)	76,045 (87.0)	17,808 (82.2)	7117 (69.6)
Any use	18,771 (15.1)	10,428 (13.0)	3858 (17.8)	3115 (30.4)
H_2_-antagonist	5924 (4.8)	3840 (3.1)	1210 (5.6)	874 (8.6)
Prostaglandins	51 (0.04)	30 (0.03)	10 (0.05)	11 (0.1)
PPI	14,160 (11.5)	8806 (9.6)	2881 (13.3)	2473 (24.2)
Other GORD drugs	882 (0.7)	543 (0.6)	167 (0.8)	172 (1.7)
Prior AI-drug use				
No use	82,021 (66.5)	65,936 (72.1)	12,281 (56.7)	3804 (37.2)
Any use	41,330 (33.5)	25,517 (27.9)	9385 (43.3)	6428 (62.8)
Corticosteroids	30,171 (24.5)	19,637 (21.5)	6681 (30.8)	3853 (37.7)
Antirheumatics	49 (0.04)	20 (0.02)	17 (0.1)	12 (0.1)
Opioids	19,588 (15.9)	10,174 (11.1)	4758 (21.9)	4656 (45.5)

Abbreviations: IQR = Inter quartile range; GI = gastrointestinal; AI = Anti-inflammatory; GORD = Drugs for peptic ulcer and gastro-esophageal reflux disease.

1 Other psychiatric: Contact with any other psychiatric disorder than the others mentioned.

2 Prior NSAID use: Use of NSAID in the year prior to antidepressant treatment initiation. It was possible to receive more than one type of NSAID. NSAIDs are classified as: Salicylates (e.g., aspirin), nonselective NSAIDs (NS-NSAIDs, e.g., ibuprofen), nonselective COX-inhibitors (e.g., diclofenac), and selective COX-inhibitors (e.g., celecoxib). In addition, prior use of paracetamol is shown.

### NSAIDs in general and paracetamol

Compared to SSRI monotherapy, NSAIDs in combination with SSRIs were associated with increased adjusted risks of any psychiatric contact and with depression only (Table 2012, Figs1 and 2). Paracetamol use decreased risks of any psychiatric contact, with depression and suicide attempts. Regarding side effects, NSAIDs increased risk of CVD contacts, but decreased risk of GI contacts (Table 2012). A more than three times increased mortality risk was observed for paracetamol in combination with SSRIs.

**Table 2:** Crude and adjusted hazard rate ratios (HRR) of treatment and safety outcomes comparing users of SSRI monotherapy with users of SSRIs in combination with NSAIDs or paracetamol

Outcome	SSRI	SSRI & NSAID	SSRI & paracetamol
*Treatment Outcomes*			
Contacts with depression			
Events	2966	224	74
Person-years	46,136.5	4612.4	2400.7
Crude HRR (95%-CI)	1.0	**1.16 (1.00; 1.34)**	**0.56 (0.43; 0.73)**
Adjusted HRR^1^ (95%-CI)	1.0	**1.31 (1.11; 1.55)**	**0.52 (0.37; 0.74)**
Psychiatric Contacts			
Events	5823	398	129
Person-years	42,710.5	4602.7	2397.1
Crude HRR (95%-CI)	1.0	1.11 (0.99; 1.24)	**0.72 (0.59; 0.88)**
Adjusted HRR^1^ (95%-CI)	1.0	**1.22 (1.07; 1.38)**	**0.56 (0.44; 0.71)**
Suicide attempts			
Events	837	40	10
Person-years	44,900.8	4624.8	2404.4
Crude HRR (95%-CI)	1.0	0.97 (0.70; 1.36)	**0.48 (0.23; 0.98)**
Adjusted HRR^1^ (95%-CI)	1.0	0.98 (0.69; 1.41)	**0.43 (0.20; 0.92)**
Committed suicide			
Events	63	8	Too few events
Person-years	45,448.3	4625.6	-
Crude HRR (95%-CI)	1.0	1.34 (0.63; 2.85)	-
Adjusted HRR^1^ (95%-CI)	1.0	0.85 (0.29; 2.50)	-
*Safety Outcomes*			
All-cause mortality			
Events	1734	492	978
Person-years	45,174.5	4588.5	2339.4
Crude HRR (95%-CI)	1.0	1.03 (0.93; 1.14)	**3.69 (3.38; 4.02)**
Adjusted HRR^1^ (95%-CI)	1.0	1.02 (0.89; 1.17)	**3.18 (2.83; 3.58)**
CVD mortality			
Events	508	182	280
Person-years	44,079.4	5595.3	3581.1
Crude HRR (95%-CI)	1.0	**1.22 (1.04; 1.44)**	**2.56 (2.20; 2.97)**
Adjusted HRR^1^ (95%-CI)	1.0	0.99 (0.75; 1.31)	**2.51 (1.93; 3.28)**
GI mortality			
Events	67	34	37
Person-years	48,186.2	5764.5	3931.7
Crude HRR (95%-CI)	1.0	1.48 (0.96; 2.28)	**3.03 (2.02; 4.54)**
Adjusted HRR^1^ (95%-CI)	1.0	1.79 (0.97; 3.31)	**2.16 (1.16; 4.00)**
CVD Contacts			
Events	1982	507	230
Person-Years	44,952.8	4579.9	3586.0
Crude HRR (95%-CI)	1.0	**1.62 (1.46; 1.81)**	1.05 (0.91; 1.21)
Adjusted HRR^1^ (95%-CI)	1.0	**1.46 (1.18; 1.67)**	1.01 (0.84; 1.21)
GI Contacts			
Events	1523	187	143
Person-Years	43,531.8	4613.5	2683.5
Crude HRR (95%-CI)	1.0	**0.84 (0.71; 0.98)**	**0.76 (0.60; 0.96)**
Adjusted HRR^1^ (95%-CI)	1.0	**0.82 (0.68; 0.99)**	1.02 (0.85; 1.22)

Abbreviations and explanations: HRR = Hazard rate ratio; 95%-CI = 95% confidence interval; NSAID = nonsteroidal anti-inflammatory drug; SSRI = Selective serotonin reuptake inhibitor; **Bold** numbers represent statistically significant results.

1 The results are adjusted for: Age; gender; educational level; previous contacts with psychiatric and somatic disorders; Charlson Index score; prior use of NSAIDs, paracetamol, other anti-inflammatory and GI-protective drugs within the preceding year to index date; SSRI start year, and earlier suicide attempts.

**Figure 1:**
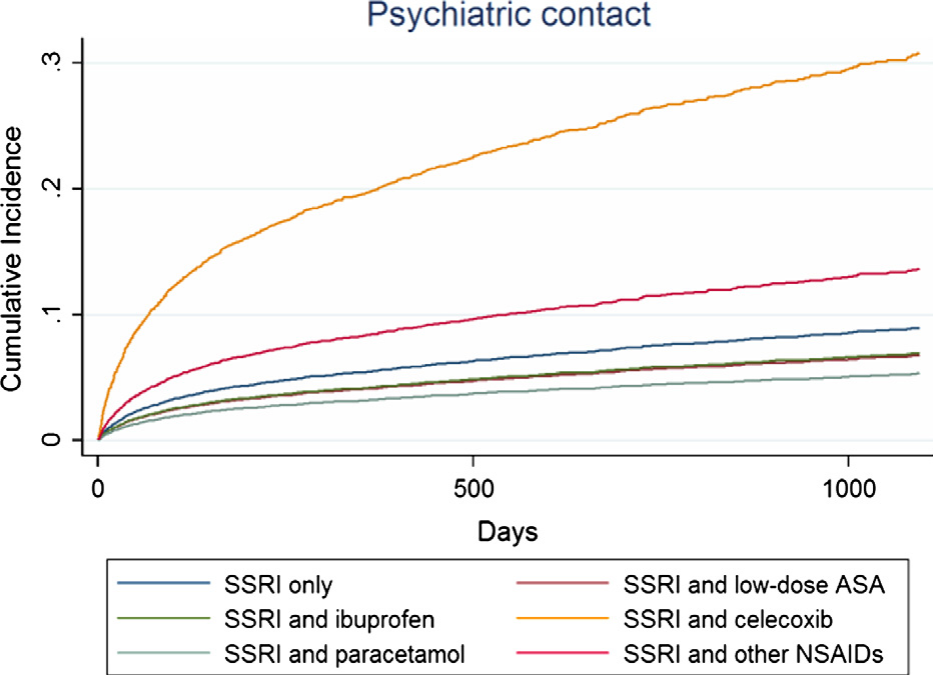
Cumulative incidences * illustrating risk of any psychiatric contact among SSRI users compared to users of SSRIs in combination with different NSAIDs or paracetamol within the first 3 years (1095 days) of follow-up.

**Figure 2:**
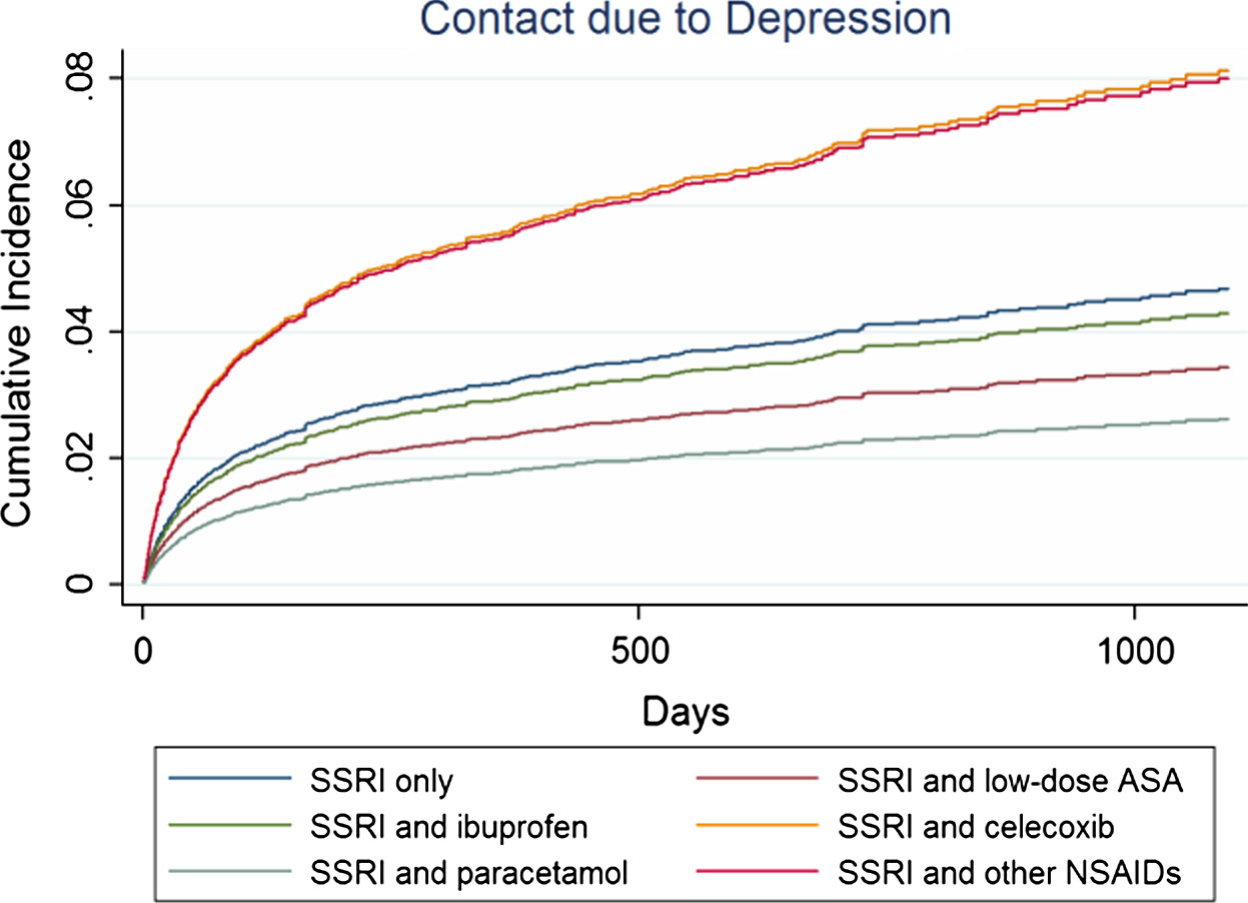
Cumulative incidences * illustrating risk of contacts with depression among SSRI users compared to users of SSRIs in combination with different NSAIDs or paracetamol within the first 3 years (1095 days) of follow-up.

Tables 2009 and 2010 display results for treatment effectiveness and for safety outcomes of the different NSAID groups and single NSAIDs. Salicylates decreased risks of any psychiatric contact and with depression, whereas both NS-COX and selective COX-2 inhibitors were associated with increased risks. NS-COX and selective COX-2 inhibitors increased mortality risk, whereas salicylates were associated with a higher risk of CVD contacts.

**Table 3:** Treatment outcomes for SSRIs in combination with the different NSAID groups and selected NSAIDs

	Contacts with depression			Any Psychiatric Contacts			Suicide Attempts		
	*N* (events)	Person-Years	Adjusted* HRR (95%-CI)	*N* (events)	Person-Years	Adjusted* HRR (95%-CI)	*N* (events)	Person-Years	Adjusted* HRR (95%-CI)
SSRI only	2966	46,136.5	1.0	5823	42,710.5	1.0	837	44,900.8	1.0
Salicylates	83	2653.8	0.77 (0.57; 1.03)	117	2651.4	**0.75 (0.59; 0.95)**	9	2662.4	0.59 (0.25; 1.40)
ASA	15	369.5	1.02 (0.55; 1.90)	16	303.7	1.03 (0.61; 1.75)	<4^	-	-
ASA low-dose	61	2184.3	0.71 (0.50; 1.01)	88	2182.2	**0.74 (0.56; 0.98)**	6	2192.2	0.38 (0.12; 1.26)
Non-sel. NSAIDs	46	1203.3	0.78 (0.56; 1.08)	137	1197.4	1.12 (0.94; 1.35)	18	1205.1	0.99 (0.60; 1.64)
Ibuprofen	43	956.1	0.92 (0.65; 1.29)	81	954.1	**0.76 (0.60; 0.98)**	15	958.0	1.07 (0.62; 1.87)
Naproxen	<4^	-	-	50	100.8	**4.70 (3.51; 6.29)**	<4^	-	-
Non-sel. COX	92	698.4	**3.31 (2.64; 4.16)**	114	697.5	**1.90 (1.53; 2.37)**	9	700.3	0.96 (0.45; 2.04)
Diclofenac	83	423.0	**4.47 (3.58; 5.59)**	99	422.2	**2.60 (2.07; 3.27)**	5	424.5	0.78 (0.29; 2.10)
Selective COX-2	11	242.5	1.33 (0.64; 2.78)	25	241.5	**2.17 (1.37; 3.44)**	5	242.9	**3.20 (1.18; 8.72)**
Celecoxib	8	113.6	1.74 (0.70; 4.30)	22	112.6	**3.84 (2.40; 6.13)**	5	114.0	**5.09 (1.65; 15.72)**

Abbreviations and explanations: HRR=Hazard rate ratio; 95%-CI=95% Confidence Interval; ASA=acetylsalicylic acid; NSAID = nonsteroidal anti-inflammatory drug; COX = Cyclooxygenase; SSRI = Selective serotonin reuptake inhibitor; **Bold** numbers represent statistically significant results.

* The results are adjusted for: Age; gender; educational level; previous contacts with psychiatric and somatic disorders; Charlson Index score; prior use of NSAIDs, paracetamol, other anti-inflammatory and GI-protective drugs within the preceding year to index date; SSRI start year and earlier suicide attempts. ^Number of less than 4 are not reportable due to data protection.

**Table 4:** Safety outcomes for SSRIs in combination with the different NSAID-groups and selected NSAIDs

	All-cause mortality			Cardiovascular mortality			Gastrointestinal mortality		
	*N* (events)	Person-Years	Adjusted* HRR (95%-CI)	*N* (events)	Person-Years	Adjusted* HRR (95%-CI)	*N* (events)	Person-Years	Adjusted* HRR (95%-CI)
SSRI only	1734	45,174.5	1.0	508	44,079.4	1.0	67	48,186.2	1.0
Salicylates	319	2636.3	0.89 (0.75; 1.06)	140	3222.4	1.18 (0.85; 1.62)	14	3252.2	0.89 (0.33; 2.39)
ASA	49	303.4	0.85 (0.56; 1.29)	25	369.3	**0.32 (0.13; 0.77)**	4	428.9	0.64 (0.09; 4.62)
ASA low-dose	262	2167.6	0.91 (0.75; 1.10)	115	2687.2	**1.58 (1.13; 2.22)**	10	2615.7	1.16 (0.40; 3.39)
Non-sel. NSAIDs	81	1201.4	1.12 (0.83; 1.51)	24	1336.9	1.09 0.56; 2.10)	5	1414.0	1.64 (0.52; 5.15)
Ibuprofen	65	955.3	1.09 (0.79; 1.51)	19	1052.6	1.19 (0.60; 2.37)	5	1083.9	1.22 (0.30; 4.90)
Non-sel. COX	87	695.7	**1.41 (1.04; 1.92)**	22	771.5	0.52 (0.19; 1.42)	10	1128.8	**3.92 (1.54; 10.00)**
Diclofenac	52	421.4	**1.77 (1.22; 2.55)**	12	456.6	0.78 (0.25; 2.47)	8	771.3	**5.48 (1.85; 16.18)**
Selective COX-2	61	238.3	**1.75 (1.21; 2.53)**	16	308.6	0.94 (0.38; 2.35)	5	446.9	1.86 (0.39; 8.88)
Celecoxib	28	111.8	**1.86 (1.15; 3.02)**	5	145.0	0.90 (0.23; 3.58)	5	235.4	4.19 (0.87; 20.15)

	CVD Contacts			GI Contacts		
	N (events)	Person-Years	Adjusted* HRR (95%-CI)	N (events)	Person-Years	Adjusted* HRR (95%-CI)
SSRI only	1982	44,952.8	1.0	1523	43,531.8	1.0
Salicylates	410	2621.6	**1.85 (1.60; 2.15)**	92	2655.7	0.79 (0.60; 1.04)
ASA	91	366.2	1.00 (0.76; 1.32)	24	329.3	0.76 (0.47; 1.23)
ASA low-dose	310	2662.4	**2.51 (2.14; 2.95)**	58	2470.1	0.86 (0.62; 1.20)
Non-sel. NSAIDs	64	1202.4	0.99 (0.73; 1.35)	57	1202.4	0.96 (0.69; 1.33)
Ibuprofen	54	1050.6	0.99 (0.71; 1.38)	43	1055.6	0.95 (0.66; 1.36)
Naproxen	6	116.9	1.47 (0.60; 3.55)	5	113.5	0.84 (0.27; 2.63)
Non-sel. COX	36	698.9	0.74 (0.49; 1.10)	39	698.7	0.96 (0.65; 1.42)
Diclofenac	18	456.6	0.75 (0.45; 1.25)	28	467.2	1.18 (0.75; 1.84)
Etodolac	7	159.5	0.72 (0.30; 1.76)	9	175.0	1.15 (0.54; 2.45)
Selective COX-2	14	242.0	**0.41 (0.19; 0.87)**	14	241.9	0.65 (0.34; 1.27)
Celecoxib	7	144.6	0.56 (0.21; 1.50)	8	125.3	0.84 (0.35; 2.04)
Rofecoxib	7	157.0	0.32 (0.10; 1.00)	6	141.0	0.55 (0.20; 1.50)

Abbreviations and explanations: HRR = Hazard rate ratio; 95%-CI = 95% Confidence Interval; ASA = acetylsalicylic acid; NSAID = nonsteroidal anti-inflammatory drug; COX = Cyclooxygenase; SSRI = Selective serotonin reuptake inhibitor; **Bold** numbers represent statistically significant results.

* The results are adjusted for: Age; gender; educational level; previous contacts with psychiatric and somatic disorders; Charlson Index score; prior use of NSAIDs, paracetamol, other anti-inflammatory and GI-protective drugs within the preceding year to index date; SSRI start year and earlier suicide attempts.

All analyses were adjusted for the presence of comorbid somatic disorders related to indications for NSAID or paracetamol use and by using the Charlson comorbidity index. Overall, the adjustment for somatic comorbidity had no major impact on the estimated HRRs for the association between concomitant use of SSRIs and NSAIDs and the investigated outcomes. This was independent of the fact if the Charlson comorbidity index was used or if the analyses were individually adjusted for all 19 diseases included in the Charlson Index. Moreover, comorbid somatic disorders had no independent impact on antidepressant treatment outcomes (results not shown), but on mortality outcomes in a dose–response relationship. For example, the risk of all-cause mortality, compared to no comorbid somatic disorder, was HRR=1.70 (1.39; 2.07) with 1 somatic disorder, HRR=3.56 (3.00; 4.21) with two somatic disorders, and HRR=4.57 (3.85; 5.44) with three or more somatic disorders.

### Single NSAIDs

**Figure 3:**
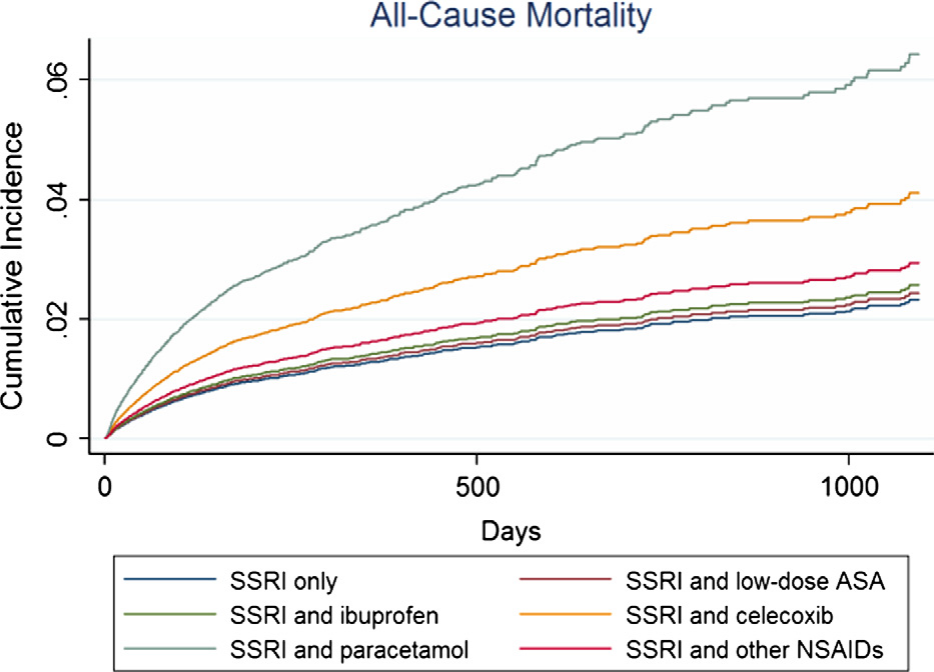
Cumulative incidences * illustrating risk of mortality among SSRI users compared to users of SSRIs in combination with different NSAIDs or paracetamol within the first 3 years (1095 days) of follow-up.

Lower risks of any psychiatric contact and for depression was observed among users of low-dose ASA (Table 2009, Figs1 and 2), whereas the risk for CVD contacts was increased. Concomitant ibuprofen decreased the risk of psychiatric contacts. Diclofenac and celecoxib were associated with significantly increased risks of psychiatric contacts and mortality, in particular GI mortality (Tables 2009 and 2010; Figs1 and 3). Diclofenac furthermore yielded a four times higher risk of contacts with depression.

### Sensitivity analyses

Mortality risks increased among users of NSAIDs in general, paracetamol and celecoxib in users younger than 60 years. All other sensitivity analyses supported results from the primary analyses.

## Discussion

This population-based cohort study on 123,351 SSRI users is the largest study to date on antidepressant treatment response and safety aspects of concomitant use of SSRIs and NSAIDs or paracetamol. We report that concomitant consumption occurs frequently on the population level. The combination therapy of SSRIs and NSAIDs in general yielded no adjunctive treatment effect with regard to psychiatric contacts in the secondary healthcare system. The investigation of individual NSAIDs, however, emphasized the heterogeneous effect of this therapeutic class; low-dose ASA and ibuprofen were associated with adjunctive treatment effects while paracetamol and the selective COX-2 inhibitors yielded an increased mortality risk.

In accordance with previous nonrandomized studies (Mendlewicz et al. 2006; Almeida et al. 2012), our results indicate that low-dose ASA could be an effective antidepressant add-on therapy to SSRIs. However, indication for prescription was not available and low-dose ASA is often prescribed prophylactic in primary or secondary prevention of cardiovascular disease, potentially resulting in better preventive care and attention in general, which partly could explain the observed effects. Clinical and animal studies support the combination therapy of SSRIs and ASA in different doses (Brunello et al. 2006; Mendlewicz et al. 2006). In individuals aged 50 or above, ASA monotherapy was associated with a reduced lifetime risk of depression (Mendlewicz et al. 2006; Almeida et al. 2012). In a recent review, the authors emphasized the antidepressant potential of ASA because of a better benefit-risk profile as compared to other anti-inflammatory drugs (Fond et al. 2013). Our results are in line with a recent study reporting that NSAIDs in general, but not ASA, worsened antidepressant treatment effects (Gallagher et al. 2012). In contrast, one study reported worse antidepressant treatment effects for ASA in any dose (Warner-Schmidt et al. 2011) and another an increase in the incidence of depression in healthy men aged 69–87 using ASA monotherapy (Almeida et al. 2010). However, the uncertainty of length of concomitant use of antidepressants and NSAIDs (Warner-Schmidt et al. 2011; Gallagher et al. 2012) may have introduced misclassification with regard to concomitant exposure.

Two new and potentially important discoveries emerged from this study. First, we showed that ibuprofen may be an adjunctive treatment approach in combination with SSRIs contrasting one prior study associating ibuprofen with inhibition of antidepressant treatment (Warner-Schmidt et al. 2011). Second, paracetamol in combination with SSRIs was related to a more than twofold increased cardiovascular mortality in our study compared to a 1.28 times increased cardiovascular mortality risk found for paracetamol monotherapy (de Vries et al. 2010). COX-2 inhibition may explain these epidemiological findings (Hinz and Brune 2012), but an earlier study emphasized the risk for confounding by indication (Lipworth et al. 2003). Although the interaction between paracetamol only and the combination with SSRIs was not addressed in the current study, our findings add concern to a possible risk for cardiovascular adverse events among users of SSRIs in combination with paracetamol despite the findings of decreased risks of any psychiatric contact, with depression and suicide attempts.

The entire group of selective COX-2 inhibitors showed an increased mortality risk and no adjunctive antidepressant treatment effects. This is in contrast to clinical studies, associating short-term celecoxib add-on treatment with antidepressant properties (Muller et al. 2006; Akhondzadeh et al. 2009; Abbasi et al. 2012) without increased CVD-risk within the first 60 days (Solomon et al. 2006). In addition, a recent study associated COX-2 inhibitors with slightly adjunctive treatment effects (Gallagher et al. 2012). The difference to our findings may originate in different aspects. The clinical trials (Muller et al. 2006; Akhondzadeh et al. 2009; Abbasi et al. 2012) studied depressed patients, whereas this study investigated SSRI users, without specific knowledge of the indication. The increased risk for suicide attempts was only based on five individuals among celecoxib users. In this study, citalopram was used by 58% as compared to different antidepressants in the clinical studies. Furthermore, in this study, half of NSAID and paracetamol users were aged 70 years or older, whereas in the clinical trials, participants were mainly between 18 and 65 years of age. Thus, other psychiatric disorders may have contributed to the increased risk for psychiatric contacts observed in the current study.

Only one study reported that use of citalopram in combination with NSAIDs and paracetamol was associated with decreased antidepressant treatment effects (Warner-Schmidt et al. 2011). The authors recommended a carefully balanced use of anti-inflammatory agents in patients suffering from depression. Our results support a more differentiated view on this topic; NSAIDs are a very heterogeneous group of drugs (Knights et al. 2010) and both treatment effects and safety aspects varied across the different NSAIDs. Depression is often comorbid with painful conditions (Manning and Jackson 2013), why many require this combination therapy.

Side effects concerning use of SSRIs and NSAIDs has been intensively investigated (de Abajo and Garcia-Rodriguez 2008; Schjerning Olsen et al. 2011). It is noteworthy, though that in this study, different NSAIDs showed different risk-profiles and commonly used NSAIDs were not associated with important CVD or GI side effects. However, confounding by indication should always be kept in mind. Low-dose ASA is used prophylactic, for example, among patients with a high risk for CVD adverse events, partly explaining the observed increased CVD risk. Celecoxib may preferably be prescribed to patients with increased GI risk-profiles, possibly explaining the observed increased GI mortality. Regarding our findings of decreased risks for GI contacts among NSAID users, we investigated if these findings depended on baseline risk factors. Previous studies that found increased risks for GI bleedings have investigated individuals without these risk factors, for example, excluding patients with cancer or prior GI bleeding events, and included more men and older individuals compared to our study (de Abajo and Garcia-Rodriguez 2008). Subanalyses of our data revealed that the decreased risk was driven by individuals with prior GI drug use: Whereas, among individuals without prior GI drug use, risk for GI contacts was increased (2.6 (95%-CI: 1.4; 4.9)). This is in accordance with previous findings of limited increased risks among users of proton pump inhibitors (de Abajo and Garcia-Rodriguez 2008).

### Strengths and limitations

We did not include users of NSAID or paracetamol monotherapy as we intended, similar to previous studies (Muller et al. 2006; Warner-Schmidt et al. 2011), to investigate the potential attenuation of antidepressant treatment effects by the combination of SSRIs with NSAIDs or paracetamol.

The strengths are the population-based design, the stable population and the validity and coverage of the registers (Helweg-Larsen 2011; Kildemoes et al. 2011; Lynge et al. 2011; Mors et al. 2011; Pedersen 2011). We investigated the entire group of NSAIDs on both effectiveness and safety measures, which is highly important because of the heterogeneity of this drug-group and as many patients rely on this combination therapy due to comorbidity (Manning and Jackson 2013).

Half of all users of the combination therapy were 70 years and older, why confounding by indication should be expected. However, several sensitivity analyses supported our results.

Though our findings concerning low-dose ASA are encouraging, they should still be interpreted cautiously as a higher CVD risk could be observed, potentially due to confounding by indication. Regarding the risk for confounding by indication, it has to be stressed that the indications for NSAIDs are generally somatic disorders, for example, pain for ASA, ibuprofen, and diclofenac and low-dose ASA for cardiovascular prevention.

Only redeemed prescriptions are registered, why it is not known whether the medications were actually consumed or other over-the-counter (OTC) medications used concomitantly. As NSAIDs are often sold OTC and compliance among antidepressant users is known to be low (Sawada et al. 2009), misclassification may have occurred. However, regarding the calculation of treatment length, the method applied in this study has been evaluated as clinically relevant (Gardarsdottir et al. 2006). Furthermore, as indication of SSRI treatment is not registered and SSRIs are used in the treatment of other disorders, such as anxiety (Donovan et al. 2010), the indication of depression is not always the case.

Due to lack of power the risk of completed suicide could only be assessed in users of NSAIDs in general and paracetamol. Furthermore, the exact cause of death may not always be available or registered correctly as autopsies are rarely performed (Helweg-Larsen 2011).

## Conclusion

Our results suggest that specific NSAIDs, such as ibuprofen and especially low-dose ASA, may imply a novel antidepressant treatment approach supporting increased efforts to conduct randomized clinical trials. While celecoxib showed adjunctive effects in randomized controlled trials, it was associated with an increased risk of mortality and psychiatric contacts in the current study. The findings concerning an increased cardiovascular mortality risk for paracetamol should be investigated further. However, all these findings have to be evaluated carefully due to the risk for confounding by indication.

The current evidence neither supports nor discourages NSAID or paracetamol add-on treatment to antidepressants, why no straight forward conclusions concerning clinical advice or guidelines can be drawn. In particular as many patients rely on the combination therapy because of the high comorbidity between depression and pain-causing disorders (Manning and Jackson 2013), which themselves can worsen depression outcomes (DeVeaugh-Geiss et al. 2010). Thus, outcomes of concomitant use of SSRIs and NSAIDs are of major concern for public health. More research is necessary to investigate which NSAIDs contain adjunctive antidepressant properties and to identify subgroups of patients that could benefit of this intervention. Regarding side effects it seems that a more differentiated view on different NSAIDs is warranted.

## Supporting Information

**Table S1.** ICD-8 and ICD-10 codes used to identify both antidepressant treatment outcomes and adverse events.
