# Supplementary material for: Inflammation and depression: combined use of selective serotonin reuptake inhibitors and NSAIDs or paracetamol and psychiatric outcomes
Source: Brain Behav. 2015 May 29;5(8):e00338. doi: 10.1002/brb3.338 (PMC4559013; doi:10.1002/brb3.338)
Supplement: Supplementary file 1 [file brb30005-e00338-sd1.docx]

**Supplementary Table 1: ICD-8 and ICD-10 codes used to identify both antidepressant treatment outcomes and adverse events.**

| Disorder | ICD-8 codes | ICD-10 codes |
| --- | --- | --- |
| Psychiatric: |  |  |
| Disorders due to use of alcohol | 291, 303, 570, 571.00, 571.10, 573.00, 573.01, 577.10, 979, 980 | F10 |
| Disorders due to use psychoactive substance use | 304, 294.4 | F11-16 and F18-19 |
| Schizophrenia spectrum | 295 | F20-29 |
| Bipolar disorder | 296.16, 296.39, 298.19 | F30-31 |
| Depression | 296.09, 296.29, 296.99 | F32-33 |
| Anxiety disorders | 300.09 | F40-42 |
| Other psychiatric  disorders | All other remaining psychiatric ICD-8 codes than the above mentioned | All other remaining psychiatric ICD-10 codes than the above mentioned |
| Suicide attempts | E953-957, 800-959 | X70-79, X80-82, S00-T32.9, T71,  T75-T75.8 |
| Committed suicide | Non applicable* | X60-84 |
| Somatic: |  |  |
| Musculoskeletal system or connective tissue disorders | 730-738, 754-756, 787 | M00-M99 |
| CVD^§^ | Non applicable* | I00-99 |
| GI^§^ | Non applicable* | K25.0, K25.2, K25.4, K25.6, K26.0, K26.2, K26.4, K26.6, K27.0, K27.2, K27.4, K27.6, K28.0, K28.2, K28.4, K28.6, K29.0, K92.0, K92.1, K92. 2 |

Abbreviations: ICD=International Classification of Diseases; CVD=Cardiovascular Disorder; GI=Gastrointestinal disorder;

*ICD-8 codes for CVD and GI events and committed suicide were not used because these outcomeswere only identified after January, 1 1996, where ICD-10 was implemented.

^§^ICD-codes for CVD and GI events were used concerning both mortality and hospital contacts.
